# Mediator Subunit 12 Is Required for Neutrophil Development in Zebrafish

**DOI:** 10.1371/journal.pone.0023845

**Published:** 2011-08-25

**Authors:** Maria-Cristina Keightley, Judith E. Layton, John W. Hayman, Joan K. Heath, Graham J. Lieschke

**Affiliations:** 1 Australian Regenerative Medicine Institute, Monash University, Clayton, Victoria, Australia; 2 Cancer and Haematology Division, Walter and Eliza Hall Institute of Medical Research, Parkville, Victoria, Australia; 3 Department of Medical Biology, University of Melbourne, Parkville, Victoria, Australia; 4 Colon Molecular and Cell Biology Laboratory, Melbourne Branch, Ludwig Institute for Cancer Research, The Royal Melbourne Hospital, Parkville, Victoria, Australia; Hong Kong University of Science and Technology, China

## Abstract

Hematopoiesis requires the spatiotemporal organization of regulatory factors to successfully orchestrate diverse lineage specificity from stem and progenitor cells. Med12 is a regulatory component of the large Mediator complex that enables contact between the general RNA polymerase II transcriptional machinery and enhancer bound regulatory factors. We have identified a new zebrafish *med12* allele, *syr*, with a single missense mutation causing a valine to aspartic acid change at position 1046. *Syr* shows defects in hematopoiesis, which predominantly affect the myeloid lineage. *Syr* has identified a hematopoietic cell-specific requirement for Med12, suggesting a new role for this transcriptional regulator.

## Introduction

Effective hematopoiesis requires the organized differentiation of multipotent stem cells and progenitor cells into a spectrum of specialized cell types. Achieving this diverse outcome is dependent on the precise timing of initiation of multiple individual cell-restricted gene expression programs. It is important, therefore, to understand how combinatorial inputs from the general RNA polymerase (RNAP) II transcriptional machinery and lineage-specific transcription factors align to specify the development of diverse cell types. Mediator is an essential component of the transcriptional machinery that regulates the activity of RNAP II by transmitting information from transcription factors bound to upstream promoter and enhancer elements to the general transcription initiation factors bound to the core promoter. It was originally described in yeast as a coactivator of RNAP II, but is evolutionarily conserved, with an equivalent complex present in mammals [1], [2]. Mammalian Mediator comprises up to 30 proteins and exists in two major forms. The core complex, Mediator, contains 25 subunits plus Med26 and is generally required as a strong coactivator of transcription. The other form, Med12-Mediator (or CDK8-Mediator), contains the same 25 subunits plus the Med12 module, consisting of Med12, Med13, CDK8 and Cyclin C. Med12-Mediator is capable of regulating transcription both positively and negatively, depending on context. The Med12 module has been shown to act as a molecular switch that shuts down activated transcription upon binding to Mediator by blocking reinitiation by RNAP II [3]. Furthermore, Med12 but not Med13 is essential for activating CDK8 kinase and can lead to transcriptional repression independently of CDK8 kinase. However, Med12 also interacts with the activation domains of SOX9 and Rta, supporting its role in transcriptional activation [4], [5]. The hedgehog signaling effector, Gli3, targets Med12 through its activation domain and has been proposed to act by reversing the transcriptional repression caused by the Med12 module [6]. In addition, Med12 has been shown to interact with the histone methyl transferase, G9A, which is responsible for the transcriptionally repressive H3K9 methylation mark [7].

The molecular mechanisms of Mediator regulatory function are not well understood. However, it appears that individual subunits of the complex interact specifically with different activators or repressors to selectively fine-tune the regulation of specific signaling pathways. For example, Med1 interacts with nuclear receptors, Med23 is required for the MAP kinase pathway [8], myc interacts with CDK8 [9] and Med15 is required for TGFβ signaling [10]. Med12 contains a leucine-rich domain, a leucine- and serine-rich (LS) domain, a PQL (proline-, glutamine-, leucine-rich) domain and an OPA (glutamine-rich) domain. SOX9, Gli3 and β-catenin all interact with the PQL domain of Med12 [4], [6], [11] and Rta interacts with two Med12 fragments that share the N-terminal region of the PQL domain in common [5]. *In vivo* evidence supporting the importance of Med12 in these pathways is provided by several animal models. Med12 null mice, which are arrested at E7.5, show a loss of canonical Wnt/β-Catenin signaling, while Med12 hypomorphic mice also display neural tube closure defects and cardiac malformations [12]. In zebrafish, several mutant alleles of *med12* have been described, all of which are embryonic lethal and predicted to be null mutants. *kohtalo*, which contains a premature stop codon in Med12, has defects in brain, neural crest and kidney development [13]. Analysis of two other *med12* alleles, *trapped* and *motionless*, with predicted truncations in the N-terminal third of the protein, has implicated Med12 coactivation of *sox9* as the basis of the neural crest defects and revealed other neuronal defects [14], [15]. Another allele, *shiri*, demonstrates a role for Med12 in the regulation of Sox32, Sox4b, Hnf-4 and Her5 in endodermal development [16]. Aside from the involvement of the PQL domain in protein-protein interactions, the functional domains of Med12 remain largely uncharacterized. It is interesting, however, that a 12 bp insertion in the C-terminal OPA domain in humans is associated with mental retardation, schizophrenia and hypothyroidism, suggesting an important function for this domain [17], [18], [19]. While cardiovascular defects have been observed in all the above Med12-deficient animal models, their hematopoietic development has not been examined.

Hematopoiesis in zebrafish, as in mammals, occurs in two stages, primitive and definitive. During primitive hematopoiesis, progenitors develop from about 12 hours post fertilization (hpf) at two anatomically separate sites: rostrally, primitive myeloid cells expressing *pu.1* appear while caudally, erythroid–specified cells expressing *gata1* develop [20]. These cells start circulating at about 26 hpf and at this time, markers of definitive hematopoiesis such as *c-myb* and *runx1* are expressed in the trunk of the embryo just ventral to the dorsal aorta [21]. This region is equivalent to the AGM (aorta–gonad–mesonephros region) in mammals and cells produced here have been shown to migrate to the ventral tail of the embryo, forming an intermediate site which seeds hematopoietic cells to the definitive hematopoietic organs, the thymus and kidney [22]. This caudal hematopoietic tissue appears to be equivalent to the mammalian fetal liver. Myeloperoxidase (*Mpx)* can be detected from about 18 hpf in the intermediate cell mass (ICM, ventral trunk and tail), before the initiation of definitive hematopoiesis, suggesting that some granulocytes may develop during the primitive phase [23]. We have identified mature granulocytes at 2 dpf by electron microscopy [24]. Interestingly, the mutant *spadetail*, which lacks trunk mesoderm due to a mutation in T-box gene 16 (*tbx16)*, contains detectable granulocytes, providing evidence that they may arise from the rostral site of hematopoiesis as well as the ICM [20].

Congenital myeloid failure occurs either in isolation, or as part of global hematopoietic failure, or as one element of a multiple system congenital defect [25]. While some of the causative genetic defects have been discovered [26], [27], many are still unknown. In an endeavor to identify new genetic requirements of myelopoiesis, a forward genetic screen was undertaken in zebrafish for mutants defective in myeloid cell development, and *syrah (syr)*, was identified as a recessive mutant with reduced myeloperoxidase (*mpx*) expression. A missense mutation in the LS region of Med12 underpins the *syr* phenotype.

## Results

### Isolation and identification of *syr* mutation

**Figure 1:**
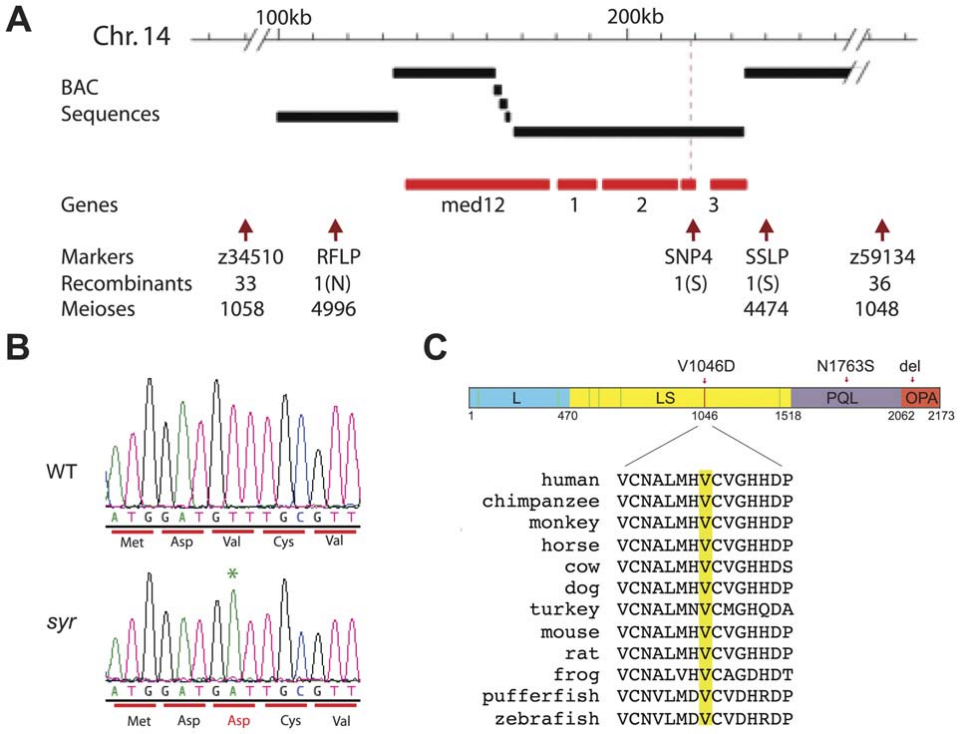
Positional cloning of *syr* gene, Med12. (A) Genetic interval showing four gene candidates: Med12, (1) Met RNA synthetase (LOC566565), (2) heatshock protein 4 (hspa4) and (3) glucosamine-6-P deaminase (gnpda1); (B) Sequencing of V1046D with mutated amino acid in red; asterisk marks the T>A transversion; (C) Schematic of Med12 protein with *syr* mutation shown by red line. Previously identified mutations are indicated by green lines; positions of N1763S and 4 amino acid deletion sequence variations are indicated; conservation of V1046 in Med12 across species (Accession numbers: XP_003209328, XP_521116, CAG08329, CAC44632, XP_002727624, CAM24448, XP_001088424, NP_005111, XP_001915364, XP_538072, XP_695002).

*Syr* was identified for its reduced expression of *mpx* at 44 hpf. The mutation is recessive and embryonic lethal, with homozygous embryos surviving for about 5 dpf. A 10 cM genome scan placed *syr* on chromosome 14 in a region dense with markers, which enabled rapid narrowing of the critical genetic interval containing the mutation (Fig. 1A). Recombinants identified in a panel of 2,785 mutant embryos narrowed the genetic interval to 4 genes, using SNP and RFLP markers that were developed in the interval. One of these genes, *med12*, was a likely candidate on the basis that another mutant of this gene displayed a combination of phenotypes similar to *syr*. cDNAs were cloned and sequenced from *syr* mutant and wild-type embryos, and a T>A transversion resulting in a V1046D amino acid change in Med12 was identified in the mutant (Fig. 1B). This residue, which lies in the LS domain in the middle of the protein, is conserved in all vertebrate sequences available and is, therefore, a likely candidate for the causative mutation (Fig. 1C). The T>A mutation was not present in the somatic DNA of the ENU-treated founder from which *syr* was derived, confirming it was ENU-induced. In addition, two sequence variations, an asparagine to serine change at amino acid 1763 (N1763S) and a 12 bp deletion at nucleotide 6374 (del) resulting in a 4 amino-acid deletion in the C-terminal OPA domain of Med12, were identified (Fig. 1C). Both of these occur in poorly conserved regions of the protein. Because the mutant is recessive, and heterozygotes do not differ from wild-type in their phenotype, *syr* is expected to be a loss-of-function mutation rather than a gain-of-function or constitutively active mutation.

### A V1046D mutation in *med12* underpins *syr*

**Figure 2:**
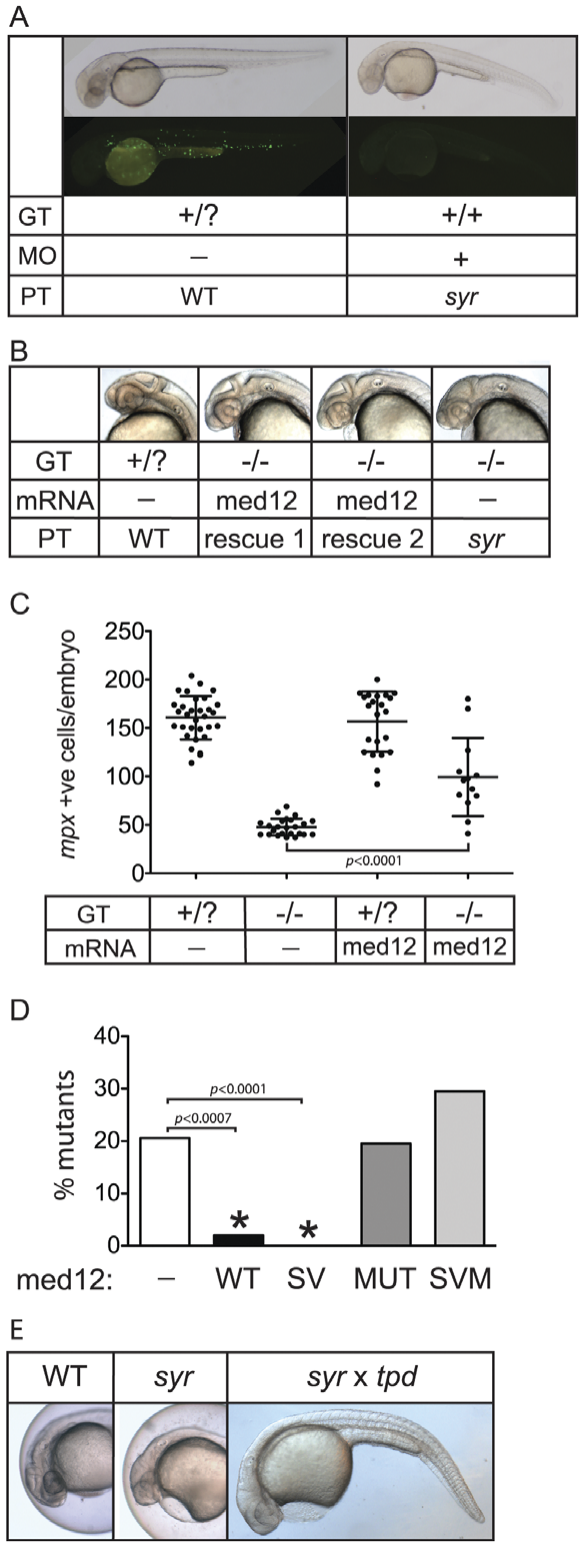
Genetic validation of V1046D mutant Med12 underpinning *syr* phenotype. (A) Phenocopy of *syr* by injection of Med12 morpholino; phenotype (PT), genotype (GT) and morpholino (MO) injection are indicated. The upper panel are bright field photos and the lower panels show WT and *syr* on a Tg(*mpx*:EGFP) background for enumeration of *mpx* expressing cells. (B) Rescue of *syr* neural phenotype with wild-type Med12; phenotype (PT), genotype (GT) and mRNA injected. (C) Rescue of myeloid defect in *syr* by overexpression of wild-type Med12 mRNA; genotype (GT) and mRNA injected are indicated. (D) Rescue of *syr* with wild-type (WT) Med12 and Med12 containing the two sequence variations, N1763S and a 12 bp deletion but not the V1046D mutation (SV). Rescue does not occur with injection of V1046D Med12 (MUT) or Med12 containing the two sequence variations in addition to the V1046D mutation (SVM). (E) Non-complementation of *syr* with *trapped (tpd)*. Rescued mutants were PCR genotype confirmed.

An amino acid change in the sequence of a highly conserved residue is probable but not conclusive as the cause of a phenotype. Based on knowledge of the phenotype, a series of genetic proofs were therefore undertaken to establish causality. Knockdown of Med12 mRNA in wild-type embryos by microinjection of a *syr* antisense morpholino oligonucleotide phenocopied the mutant in both neural and hematopoietic phenotypes (Fig. 2A). Conversely, overexpression of Med12 mRNA was able to robustly rescue both the neural and hematopoietic (decreased *mpx* expression) phenotypes of *syr* (Fig. 2B–C). There was no apparent overexpression phenotype in injected wild-type embryos. In addition to the valine to aspartic acid change at position 1046, two other sequence variations were identified: an asparagine to serine change at position 1763, and a 12 bp deletion resulting in a 4 amino acid deletion in the C-terminal OPA domain (Fig. 1C). Microinjection of Med12 mRNA containing both these sequence variations (SV), but not the V1046D mutation, fully rescued *syr* (Fig. 2D), demonstrating that these variations do not contribute to *syr*. Conversely, Med12 mRNA containing either the V1046D mutation alone or in conjunction with the two sequence variations (SVM) was not able to rescue, confirming that V1046D is the underlying mutation in *syr*. Lastly, a complementation test with another Med12 mutant allele, *tpd^t25870^* [13], showed non-complementation of the *syr* phenotype, again confirming Med12 as the gene containing the lesion responsible for *syr* (Fig. 2E).

### Med12 is required for myelopoiesis

**Figure 3:**
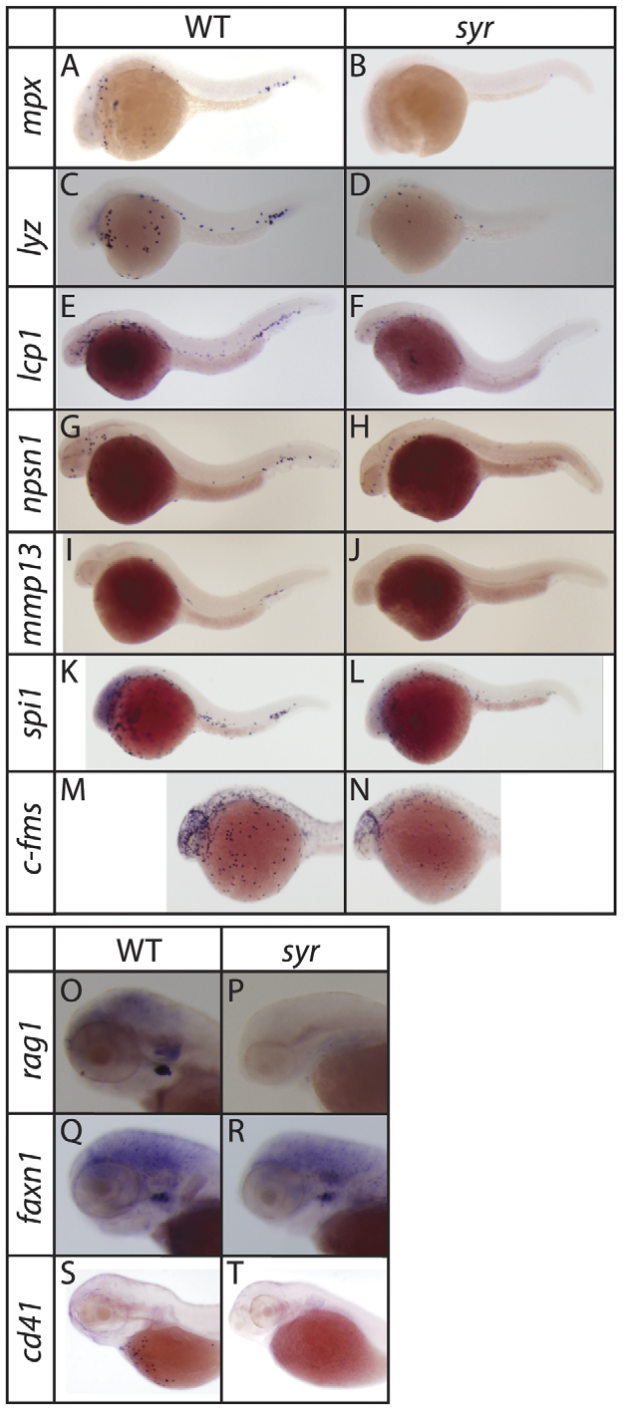
Hematopoiesis defects in *syr*. Myelomonocytic markers were examined at 23–28 hpf by WISH (A–N); T lymphocyte and thymic epithelium markers were examined at 3.5 dpf in *syr* and wt (O–R); staining of the thrombocyte marker, cd41 at 3 dpf (S–T). WISH embryos are representative of ≥4 (median = 21) examples.

Whole mount in situ hybridisation (WISH) shows reduction of *mpx* expression in *syr* is evident as early as 28 hpf, indicating either a decrease in transcription of myeloperoxidase or a decrease in cells expressing this marker. Concomitant decreases in the expression of several other myelomonocytic markers at 28–33 hpf including *lyz*, *npsn1*, *lcp1*, *spi1*, *c-fms* and *mmp13* suggest the decrease in *mpx* is likely due to a reduction in the number of neutrophils (Fig. 3A–N). In addition to myeloid markers, the expression of markers identifying the other hematopoietic lineages, were examined. Lack of *rag1* expression at 3.5 dpf suggests that T lymphocyte development may be defective (Fig. 3O–P). To distinguish between loss of thymocytes and loss of the thymus itself, f*oxn1* expression was examined (Fig. 3Q–R). Thymic epithelial cells marked by expression of *foxn1* were evident in both wild-type and mutant, suggesting the thymus is intact and that the absence of *rag1* reflects a defect or delay in thymocyte development. The thrombocyte marker, *cd41*, is also absent at 3.5 dpf in *syr* (Fig. 3S–T) pointing to an additional defect in the development of this lineage. These data from later time points are presented with the caveat, however, that since *syr* is embryonic lethal, not surviving past day 5, altered expression of these later markers could be attributed to general biological deterioration of the embryo.

### Erythropoiesis is not affected in *syr*

**Figure 4:**
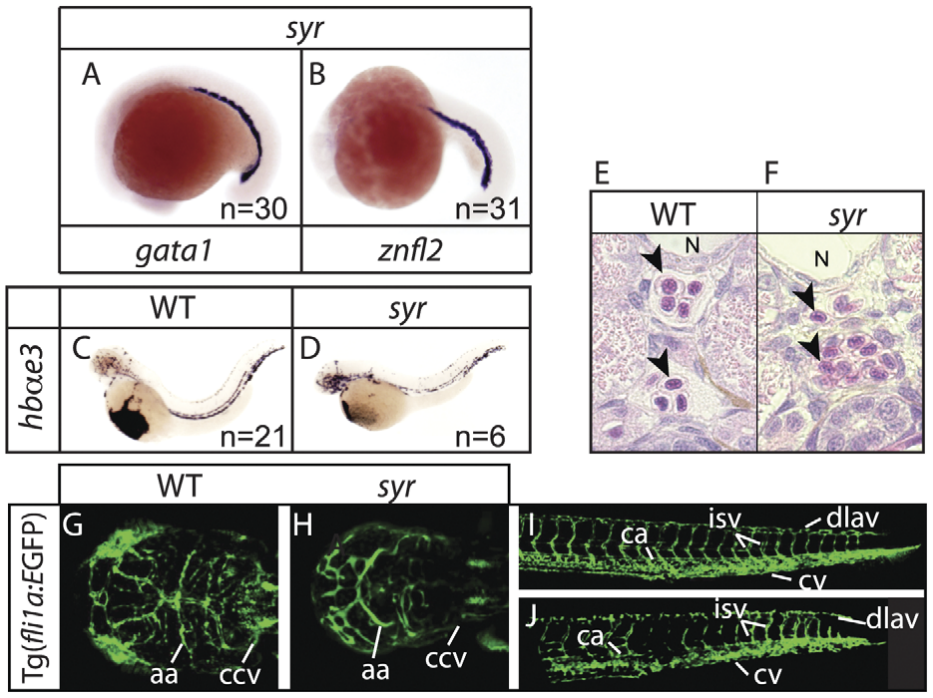
Erythropoiesis proceeds normally in *syr*. Examination of early erythroid markers at 17–19 hpf in *syr* (A–B); staining of embryonic globin in wt and *syr* at 48 hpf (C–D); transverse sections of WT and *syr* embryos at 3 dpf, counterstained with hematoxylin and eosin; notochord (N) and neutrophils (arrowheads) are indicated (E–F); *syr* crossed with Tg(*fli1a*:EGFP) shows close to normal vasculature in both head (G–H) and tail (I–J). Heads are dorsal view with anterior to left, WT on the left (G) and *syr* on the right (H); aa (aortic arches) and ccv (common cardinal vein) are indicated. Tails are lateral view, anterior to left and ca (caudal artery), cv (caudal vein), dlav (dorsal longitudinal anastomotic vessel) and isv (intersomitic vessels) are indicated.

In contrast to myeloid markers, expression of embryonic globins at 48 hpf is normal in *syr*, as is expression of two earlier erythroid markers, *gata1* and z*nfl2* at 18–20 somites. This indicates that within hematopoiesis the myeloid lineage is more affected by the *syr* defect than the erythroid lineage (Fig. 4A–D). Transverse sections through the trunk of wild-type and *syr* embryos at 3 dpf demonstrate the presence of erythrocytes (Fig. 4E–F). Although erythrocytes are seen to circulate in *syr*, there are variable circulatory defects, with a generally later onset of circulation, development of edema over the yolk and eventual cessation of circulation by 3 dpf. To determine if this resulted from defects in vascular development, *syr* was crossed with homozygous Tg(*fli1a*:EGFP)^y1^ fish, generating mutants with fluorescent vasculature. Confocal microscopy shows the head vasculature is markedly reduced and disrupted in *syr* compared to wild-type, especially in the common cardinal vein (ccv), which is difficult to see in *syr* and the aortic arches (aa) which are anteriorly bowed compared to wild-type (Fig. 4G–H). This is likely secondary to the gross abnormalities in brain morphogenesis that are noticeable from ∼28 hpf, rather than abnormality in vascular development per se. Vasculature in the tail shows formation of the major caudal artery (ca) and vein (cv) as well as the dorsal longitudinal anastomotic vessel (dlav) and intersomitic vessels (isv) in *syr* with few gross morphological differences compared to wild-type, suggesting that the cardiovascular phenotype in *syr* is not due to gross disruption of the vasculature (Fig. 4I–J).

### Med12 is required later in hematopoietic development

**Figure 5:**
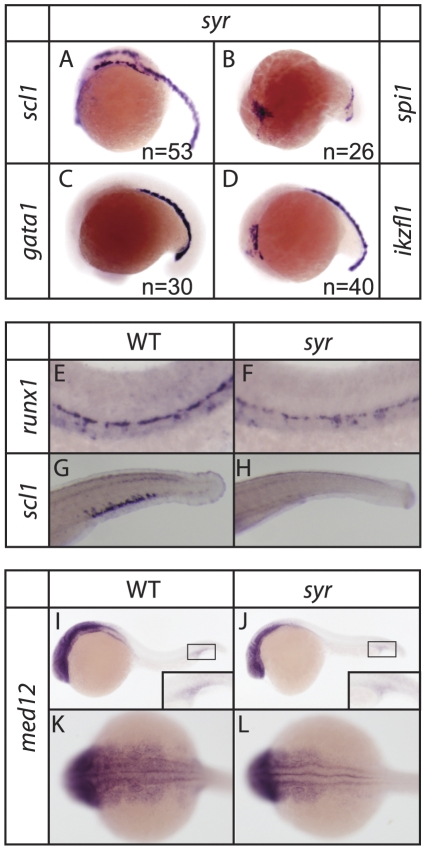
Stages of hematopoiesis in *syr*. Early hematopoietic markers are expressed normally in *syr* at 17–19 hpf (A–D) (note that Figs. 4A and 5C are the same; Fig. 4A/5C is included here for completeness). Definitive hematopoiesis is initiated in *syr* indicated by *runx1* expression at 28 hpf (E–F). Caudal hematopoietic tissue fails to develop in *syr* indicated by lack of *scl1* expression at 3 dpf (G–H). Med12 is expressed in WT and *syr* with insets showing staining in the hematopoietic ICM region (I–J); dorsal view of staining (K–L). Unless otherwise stated, WISH embryos are representative of ≥10, and ≤46, examples.

Assessment of hematopoietic development by WISH shows that in *syr* early hematopoietic progenitors are specified normally, indicated by *scl*, *pu.1*, *ikaros* and *gata1* expression comparable to wild-type at 17–19 hpf (Fig. 5A–D). The presence of *runx1* at 30 hpf in both mutant and wild-type indicates that definitive hematopoiesis is initiated (Fig. 5E–F). However, expression of *scl* is no longer evident by 3 dpf suggesting a potential defect in hematopoietic stem cell (HSC) migration from the dorsal aorta and/or failure in caudal hematopoietic tissue development (Fig. 5G–H).

In order to determine if loss of function in *syr* was due to a loss of Med12 cleared by mRNA surveillance processing, Med12 expression was analyzed. WISH showed that *Med12* was strongly expressed in the anterior of mutant as well as wild-type embryos (Fig. 5I–L) indicating *syr* has impaired Med12 function rather than a loss of transcripts. The insets of Fig. 5I–J, show Med12 localization in the ICM hematopoietic region.

### Residual neutrophils in Med12-deficient embryos can migrate

**Figure 6:**
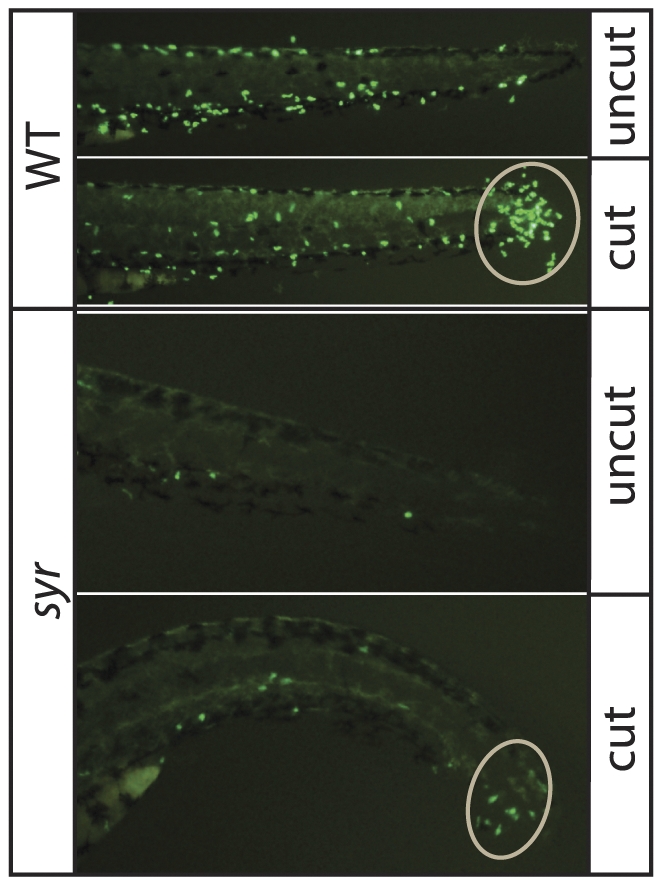
Residual neutrophils can migrate in *syr*. Top panels: WT prior to tail snip (uncut) and 8 h post transection (cut); Bottom panels: *Syr* prior to tail snip (uncut) and 8 h post transection (cut). Neutrophils at the wound margin have been circled.

A basic function of neutrophils is to migrate to the site of a wound. In order to examine whether the few neutrophils present in *syr* were capable of this behavior, a classic tail snip assay [24] was used. *Syr* was crossed onto a Tg(*mpx*:EGFP) background to facilitate visualization of leukocytes. 8 h post transection, migration of *mpx* positive cells to the wound margin was evident in both wild-type and *syr* embryos indicating that residual neutrophils can migrate in *syr* (Fig. 6).

## Discussion

A forward genetic screen has identified Med12 as critical for early myelopoiesis in zebrafish. Although several mutant alleles of Med12 have been identified, none have described the predominantly myeloid defects in hematopoiesis seen in the new *syr* allele. We have not determined the mechanism whereby these defects occur nor whether these hematopoiesis phenotypic features are direct or indirect consequences of Med12 deficiency. Primitive hematopoiesis can take place normally in *syr* but correct lineage specification does not occur and definitive hematopoiesis is compromised.

The mutation underpinning *syr* is a single T>A transversion resulting in a valine to aspartic acid change in the LS domain of Med12. This component of Mediator is highly conserved among vertebrates. The human sequence is 97% identical to mouse and 75% identical to zebrafish predicted sequences. Although the C-terminal PQL domain has been shown to be necessary for protein-protein interaction, the function of other Med12 domains remains largely unexplored. The zebrafish Med12 alleles studied previously are either nonsense mutations or mutations causing downstream nonsense mutations predicting severely truncated proteins. In contrast, *syr* is a missense mutation where Med12 transcript expression by WISH is largely indistinguishable between wild-type and mutant. This provides some insight into the role of the LS domain in Med12 function, since alteration of a single amino acid in this domain disrupts protein function.

Studies of Med12 function have identified important roles in neural, ear and endoderm development [12], [13], [14], [15], [16] as well as hepatocyte differentiation [16]. Interaction of *med1/trap220* with *gata1* has been reported, providing a precedent for the involvement of Mediator in hematopoiesis [28]. More recently, Med12/13 but not CycC or Cdk8 components of the Med12-module have been shown to be essential for transactivation in cell culture by the GATA/RUNX factors, Serpent and Lozenge, in Drosophila. Furthermore, while the Med12 module controls proliferation of the crystal cell lineage, only Med12/13 regulate its differentiation [29] suggesting a specific role for Med12 rather than a more generalized role as a component of Med. In addition, large scale microarray analysis of gene expression in the normal physiological state in human and mouse tissues and cell lines shows highest expression of Med12 in mouse B and T lymphocytes, lymph node and thymus (>3-fold above the median), and was above the median in bone marrow but below the median in spleen [29], [30]. This suggests that the absence of thymocytes in *syr* may not be due to deterioration of the embryo at this stage (3–5dpf) and that Med12 could have a role in lymphopoiesis. Because *syr* embryos do not survive long enough for B cell development to occur, this pathway has not been investigated. The human results were similar, with high expression in lymphocytes, peripheral blood CD14+ monocytes and CD34+ BM cells. These results are consistent with the expression we have seen in the zebrafish ICM (data not shown). There is also evidence for differential Med12 protein expression from the Swedish human protein database [31] and Human Protein Atlas (www.proteinatlas.org/ ENSG00000184634; accessed 6/23/11). Immunohistochemistry data from one antibody shows greatest expression in bone marrow, placenta and Purkinje cells while a stronger antibody was less discriminating, showing expression in most tissues. These data are consistent with the hypothesis that *Med12* may act in hematopoiesis by a cell autonomous mechanism and also provide evidence for a role of *Med12* in hematopoiesis independent of the *syr* mutant.

There is in vitro evidence to suggest that myc interacts with CDK8 [9] and because of the role of myc in hematopoiesis and disease it has been considered a potential target for Med12 [32], [33]. We considered the *syr* phenotype could be due in part to defective transcription of *myc*, but though expression of mych, a member of the myc family in zebrafish that is expressed in a subpopulation of neutrophils [34], appears to be decreased in *syr* compared to wild-type, this is most likely due to fewer leukocytes in *syr* rather than an actual decrease in mych transcripts. Similarly, microinjection of *mych* did not rescue *syr* (data not shown). This *in vivo* observation confirms the *in vitro* dissection of the myc-Mediator interaction, which indicates that CDK8 but not Med12 or Med13 are able to interact with myc [35].

There is growing evidence that loss of ubiquitous proteins such as hspa9b, ribosomal protein Rps19, TIF1Υ and cpsf1 can selectively affect hematopoiesis [36], [37], [38], [39]. The overarching function of Mediator is to orchestrate spatiotemporal localization of regulatory factors so that proteins bound to the enhancer can be brought into contact with transcriptional machinery at the promoter [1], [2], [40]. Recently, Mediator and cohesin have been shown to be localized uniquely at active, cell type-specific genes, forming a set of DNA loops specific to each cell type [41]. We therefore propose that the missense mutation in Med12 could potentially affect direct or indirect interaction with required hematopoietic-specific factors or disrupt the DNA loop signature of myeloid genes to generate the hematopoietic defects seen in *syr*.

## Materials and Methods

### Ethics Statement

Fish were housed in the Ludwig Institute for Cancer Research Aquarium using standard husbandry practices. All experiments were approved by the Ludwig Institute for Cancer Research or Walter and Eliza Hall Institute Animal Ethics Committees (AEC Approval IDs 2007.012 and 2009.027).

### Zebrafish lines

Zebrafish strains used were: AB*, *syrah* (*syr* ^gl10^, a novel mutant isolated in our ethylnitrosourea mutagenesis screen [42] for its lack of myeloperoxidase [*mpx*] expression), Tg(*fli1a*:EGFP)^y1^ [43] and Tg(*mpx*:EGFP)^i114^ [44]. Zebrafish gene, protein, and mutant naming follow the nomenclature conventions recommended by www.zfin.org.

### Microinjections

Fertilized 1- to 2-cell embryos were microinjected with 1 to 2 nL synthetic mRNA (520 ng/µL in H_2_O), Med12 splice site morpholino oligonucleotide [14] (350 µmol/L in H_2_O; Gene Tools, Philomath, OR); or control MO (see Table S1 for sequence), (130 µmol/L in H_2_O; Gene_Tools) traced where appropriate by mixing 1∶1 with 5% rhodamine-dextran (in 0.2 mol/L KCl).

### Positional cloning, oligonucleotides and constructs

A 10 cM scan was performed against a panel of SSLP (simple sequence length polymorphism) markers, which placed *syr* on chromosome 14. The region was subsequently narrowed using RFLP markers and SNP analysis. Table S1 lists oligonucleotide sequences used. Sequence data have been deposited in GenBank (Accession number: JN226108). Med12 constructs were derived from pCS2+ or pBluescript II KS+ (Thermo Fisher Scientific, Australia) by standard cloning techniques and linearized with NotI for in vitro transcription of capped mRNA using the mMESSAGE mMACHINE kit (Ambion, Austin, TX). Internal AflII and MluI restriction endonuclease sites were used to isolate the V1046D mutation from the C-terminal N1763S and12 bp deletion mutations and vice versa using a region swapping approach (see Table S1 for cloning primers). All constructs were validated by sequencing.

### Gene expression analysis

Whole mount in situ hybridization (WISH) was performed using standard techniques [20]. In vitro transcribed digoxigenin- or fluorescein-labeled antisense *cd41*, *c-fms*, *foxn1*, *gata1*, *hbae3*, *ikzfl1*, *lcp1*, *lyz*, *mmp13*, *mpx*, *npsn1*, *rag1*, *runx1*, *scl1*, *spi1* and *znfl2* riboprobes were detected with 4-nitroblue tetrazolium/5-bromo-4-chloro-3-indolyl phosphate.

### Imaging

Low-power images were collected using a Nikon SMZ1500 or Nikon 90i fluorescence microscope equipped with a DXM1200c camera and Nis-Elements AR software (Nikon, Tokyo, Japan), and high-power images with a Nikon Optiphot-2 microscope with a Zeiss AxioCam MRc5 digital camera and AxioVision AC (Release 4.5) software (Zeiss, Welwyn Garden City, United Kingdom). Images were imported into Adobe Photoshop CS2 9.0.2 or Illustrator CS2 12.0.1 (Adobe Systems, Mountain View, CA) for orientation and figure preparation.

### Tail snip assay

Tails of 6 to 8 embryonic zebrafish were transected at 49 hpf as described [24]. 8 h post-transection, embryos were examined for localization of EGFP-marked leukocytes.

### Genotyping

*syr* embryos were recognized as a Mendelian proportion and by their characteristic syndrome of small eyes, disruption of neural region, lack of ventricle inflation, and thinner yolk extension. Younger *syr* embryos were PCR-genotyped at the closely linked simple sequence length polymorphism (SSLP) marker z7495 (oligonucleotides, Table S1; 10 µL reactions; Phusion polymerase [New England Biolabs, Ipswich, MA] with supplied GC buffer; 92°C, 2 minutes followed by 40 cycles at 92°C, 60°C, and 72°C for 30, 30, and 60 seconds, respectively; PCR products were separated by 2% agarose gel electrophoresis or sequenced following PCR.

## Supporting Information

Table S1 **Sequences of oligonucleotides used in this study.**
(TIF)
