# Supplementary figures and images for: Mediator Subunit 12 Is Required for Neutrophil Development in Zebrafish
Source: PLoS One. 2011 Aug 25;6(8):e23845. doi: 10.1371/journal.pone.0023845 (PMC3162013; doi:10.1371/journal.pone.0023845)

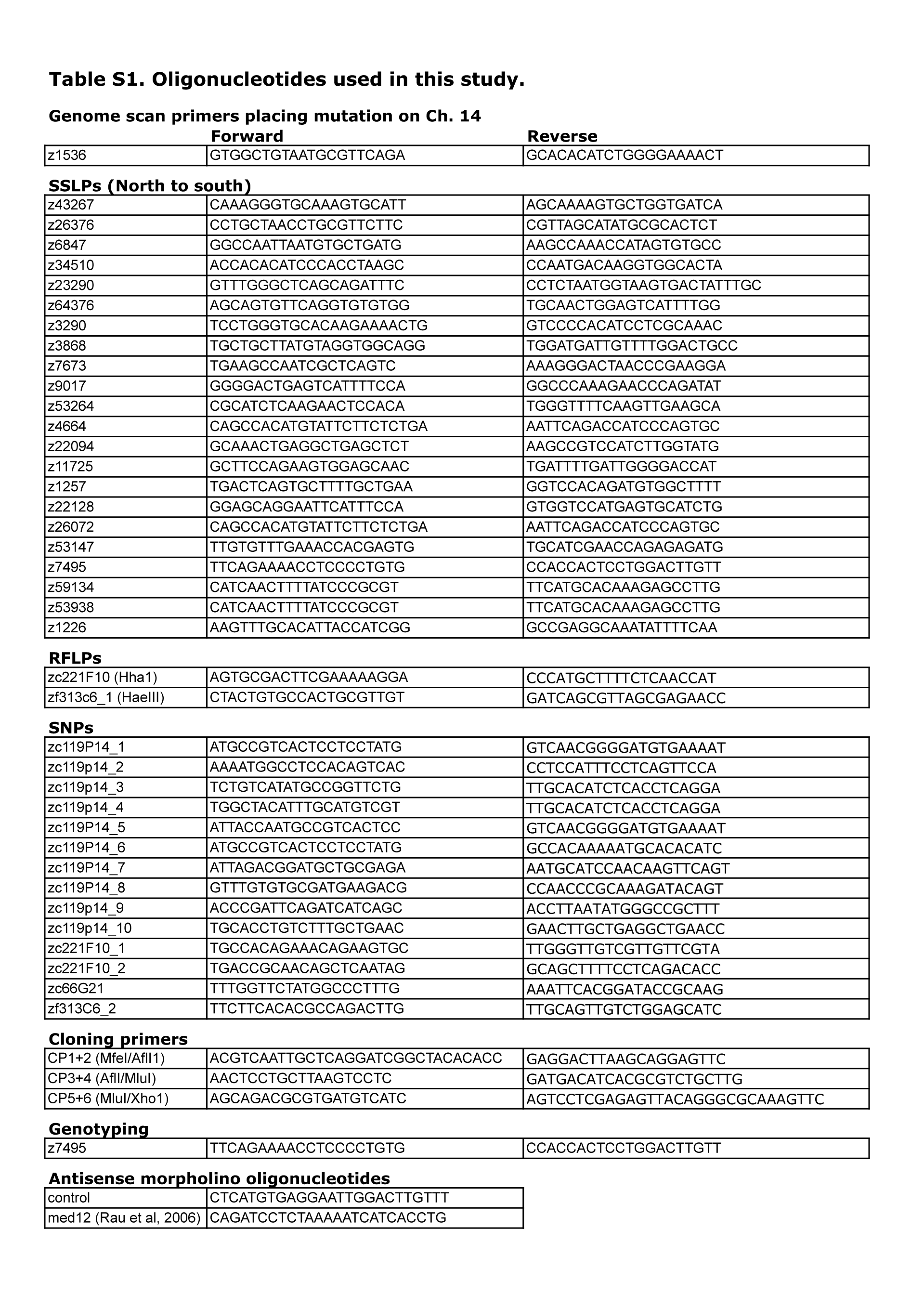

Supplement: Table S1 — Sequences of oligonucleotides used in this study. (TIF) [file pone.0023845.s001.tif]
